# Global Phosphotyrosine Proteomics Identifies PKCδ as a Marker of Responsiveness to Src Inhibition in Colorectal Cancer

**DOI:** 10.1371/journal.pone.0080207

**Published:** 2013-11-08

**Authors:** Eliot T. McKinley, Huiling Liu, W. Hayes McDonald, Weifeng Luo, Ping Zhao, Robert J. Coffey, Steven K. Hanks, H. Charles Manning

**Affiliations:** 1 Vanderbilt University Institute of Imaging Science, Vanderbilt University, Nashville, Tennessee, United States of America; 2 Department of Biomedical Engineering, Vanderbilt University, Nashville, Tennessee, United States of America; 3 Department of Cell and Developmental Biology, Vanderbilt University School of Medicine, Nashville, Tennessee, United States of America; 4 Department of Biochemistry, Vanderbilt University, Nashville, Tennessee, United States of America; 5 Department of Medicine, Vanderbilt University School of Medicine, Nashville, Tennessee, United States of America; 6 Vanderbilt Ingram Cancer Center, Vanderbilt University Medical Center, Nashville, Tennessee, United States of America; 7 Program in Chemical and Physical Biology, Vanderbilt University School of Medicine, Nashville, Tennessee, United States of America; 8 Department of Neurosurgery, Vanderbilt University Medical Center, Nashville, Tennessee, United States of America; 9 Department of Radiology and Radiological Sciences, Vanderbilt University Medical Center, Nashville, Tennessee, United States of America;; Hungarian Academy of Sciences, Hungary

## Abstract

Sensitive and specific biomarkers of protein kinase inhibition can be leveraged to accelerate drug development studies in oncology by associating early molecular responses with target inhibition. In this study, we utilized unbiased shotgun phosphotyrosine (pY) proteomics to discover novel biomarkers of response to dasatinib, a small molecule Src-selective inhibitor, in preclinical models of colorectal cancer (CRC). We performed unbiased mass spectrometry shotgun pY proteomics to reveal the pY proteome of cultured HCT-116 colonic carcinoma cells, and then extended this analysis to HCT-116 xenograft tumors to identify pY biomarkers of dasatinib-responsiveness in vivo. Major dasatinib-responsive pY sites in xenograft tumors included sites on delta-type protein kinase C (PKCδ), CUB-domain-containing protein 1 (CDCP1), Type-II SH2-domain-containing inositol 5-phosphatase (SHIP2), and receptor protein-tyrosine phosphatase alpha (RPTPα). The pY313 site PKCδ was further supported as a relevant biomarker of dasatinib-mediated Src inhibition in HCT-116 xenografts by immunohistochemistry and immunoblotting with a phosphospecific antibody. Reduction of PKCδ pY313 was further correlated with dasatinib-mediated inhibition of Src and diminished growth as spheroids of a panel of human CRC cell lines. These studies reveal PKCδ pY313 as a promising readout of Src inhibition in CRC and potentially other solid tumors and may reflect responsiveness to dasatinib in a subset of colorectal cancers.

## Introduction

Tyrosine phosphorylation is a key signaling mechanism regulating central aspects of mammalian cell behavior including proliferation, motility, metabolism, and differentiation [1]. Protein tyrosine kinases were first recognized as products of viral oncogenes including v-src and v-abl, and as receptors for growth factors including EGF. Aberrant signaling by many of the ninety conventional tyrosine kinases encoded by the human genome has been linked to disease processes, including the development and spread of cancer [1,2]. 

Targeted therapy with tyrosine kinase inhibitors (TKIs) is an ever-expanding modality that enables personalized cancer therapy [3,4]. Landmark examples include the small molecule inhibitor imatinib that effectively treats chronic myelogenous leukemia driven by the BCR-ABL oncoprotein [5,6] as well as therapies to inhibit mutant BRAF in cancers such as melanoma [7,8]. Small molecule TKIs and neutralizing monoclonal antibodies that target the EGF receptor (EGFR) and/or the closely related ERBB2 (HER2/neu) have had success in treatment of non-small cell lung carcinoma and breast carcinoma [9,10]. 

In colorectal carcinoma (CRC), a large majority of cases display elevated activity of Src-family nonreceptor tyrosine kinases [11,12], which progressively increase in activity as tumors progress to metastatic disease [13]. Aberrant Src activity can contribute to malignancy by impacting multiple receptor systems including cadherin-mediated cell-cell junctions, integrin-mediated cell-ECM adhesions, and activated receptor complexes including EGFR [14-16]. Elevated Src activity in CRC predicts poor clinical prognosis [17]. Accordingly, there has been considerable interest in Src as a therapeutic target in CRC and other cancers [18-21]. Dasatinib, the most clinically studied Src-selective inhibitor, is an effective cytostatic agent inhibiting tumor growth, invasion, and metastasis [22]. In addition to Src-family kinases, dasatinib potently inhibits BCR-ABL and was recently shown to be superior to imatinib as a therapy for chronic myelogenous leukemia [23].

In evaluating targeted TKIs in clinical oncology, there is a need to identify relevant biomarkers that can be used to guide dose selection in preclinical development and to monitor anti-tumor activity in clinical trials. Biomarkers may also be of value in predicting whether a patient is likely to benefit from a particular treatment. Several studies have utilized varied approaches in an attempt to identify such markers [24-26]. Rationally, such biomarkers could also be specific tyrosine sites that are phosphorylated by the kinase(s) being inhibited. Thus, it is of interest to characterize the tyrosine kinase signaling pathways operating in tumor cells. Tyrosine phosphorylation in tumor cells can be systematically and comprehensively profiled using mass spectrometry to analyze peptides enriched for phosphotyrosine (pY) by immunoaffinity [27]. We have previously applied this unbiased shotgun proteomics approach to obtain an in-depth analysis of tyrosine phosphorylation in normal versus Src-transformed mouse fibroblasts, thereby characterizing the global impact of oncogenic Src [28]. In another application of this approach, pY signaling in a large sampling of non-small cell lung cancer cell lines and solid tumors revealed activated tyrosine kinases [29].

The objectives of the present study were to use shotgun pY proteomics to obtain a global view of tyrosine phosphorylation in the well-known HCT-116 human colon adenocarcinoma cell line, and to extend the analysis to HCT-116 xenograft tumors treated with dasatinib to identify dasatinib-responsive pY biomarkers. We identified pY sites on signaling proteins including PKCδ CDCP1, and RPTPα as major dasatinib-responsive sites in HCT-116 xenograft tumors that may be useful as predictive biomarkers of SRC inhibition. Finally, using spheroid cultures established from a number of human CRC cell lines, we observed a correlation between datatinib-mediated inhibition of proliferation and reduction of PKCδ pY313. Our results reveal PKCδ pY313 as a candidate biomarker for predicting response to dasatinib in CRC.

## Materials and Methods

### Cell culture and drug treatment

HCT-116 (ATCC CCL-247), Caco-2 (ATCC HTB37), Colo205 (ATCC CCL-222), DKO-1, DLD-1 (ATCC CCL-221) were obtained from ATCC and Lim1215 cells [30] were obtained from Robert Whitehead, Ludwig Institute for Cancer Research. The human CRC cell lines were maintained as a monolayer culture at subconfluent density in a 5% CO_2_, 37°C atmosphere. Growth medium consisted of Dulbecco’s Modified Eagle’s Medium (DMEM; Mediatech) for all cell lines except Colo205 which was grown in RPMI (Cellgro). Growth media was supplemented with 10% fetal bovine serum (Atlanta Biologicals), 1% antimycotic antibiotics (Mediatech), and 100 μM non-essential amino acids (Gibco-Invitrogen). All cells were passaged using 0.25% EDTA-trypsin (Gibco). 

Dasatinib (BMS-354825) was kindly provided by Richard Smykla from Bristol-Myers Squibb Oncology (Princeton, NJ). Dasatinib was dissolved in dimethyl sulfoxide (DMSO) in a 10mM stock solution. HCT-116, Caco-2, Colo205, DKO-1, and DLD-1 cells were treated with escalating dasatinib concentrations (0, 10, 100, 1000, 5000 nM) for 24 h. At the end of treatment, culture medium was removed and cells were harvested for analysis.

### Animal model

All studies were approved by the Vanderbilt University Institutional Animal Care and Use Committee and all efforts were made to minimize animal suffering. HCT-116 xenografts were generated in athymic nude mice (Harlan Sprague-Dawley) following subcutaneous injection of 2-4×10^6^ cells. Palpable tumors were detected within 2 to 4 weeks. Dasatinib was reconstituted in DMSO. Immediately prior to treatment, nine parts sterile saline was added to the drug solution. Tumor-bearing mice (0.5-1 cm longest dimension) were administered 100 μL dasatinib (60 mg/kg) or DMSO/saline vehicle by oral gavage. Animals were given either a single dose or treated daily for 5 consecutive days. For each treatment regimen, cohorts of animals were sacrificed and tumors were excised 4 h and 24 h following the final drug administration.

### Preparation of phosphotyrosine-enriched peptide samples

 Tryptic peptides enriched in phosphotyrosine were prepared from HCT-116 monolayer cultures (exponentially growing at approximately 70% confluency) and from HCT-116 xenograft tumors. Cell lysis, protein digestion, and immunoaffinity enrichment for pY-containing tryptic peptides was achieved using the PhosphoScan Kit (P-Tyr-100; Cell Signaling Technology) according to the manufacturer's protocol. For cultured cells, each round of analysis was performed using 60 mg total cellular protein (obtained from eight 150 mm dishes). Protein concentration was determined using BCA (Thermo Scientific), with bovine serum albumin (BSA; Sigma) as a standard. Tumor lysates were prepared by homogenization on ice to disperse the frozen tissue (approximately 400 mg tumor wet weight in 9 mL in PhosphoScan Lysis Buffer), followed by brief sonication and centrifugation (20,000 × *g* for 15 min) to clear insoluble material. Cleared tumor lysates containing approximately 40 mg protein (as determined by BCA assay) were used in the analysis. Tryptic peptides were generated by treating lysates overnight at 25°C with trypsin-TPCK (60:1 cellular protein:trypsin). 

### Mass spectrometry

LC-MS/MS analysis of the peptides was performed using a hybrid linear ion trap/orbitrap instrument (LTQ-Orbitrap, Thermo-Fisher) equipped with an Eksigent NanoLC-AS1 Autosampler, Eksigent NanoLC-1D plus high performance liquid chromatography (HPLC) pump (Eksigent), nano-electrospray source, and Xcalibur instrument control software. Peptides were separated on a fused silica capillary column (100 μm × 18 cm) packed with reversed phase resin (Jupiter C18, 3 μm, 300 Å; Phenomenex), and coupled with an in-line, fritted (generated with liquid silicate Kasil) trapping column (100 μm × 4 cm) also packed with C18 resin (Jupiter C18, 5 μm, 300 Å, Phenomenex) [31]. Biphasic gradient chromatographic elution was employed whereby mobile phase A consisted of 0.1% formic acid and mobile phase B consisted of 0.1% formic acid in acetonitrile. The flow rate during the loading and desalting phase of the gradient was 1.5 μL/min and during separation phase was 500 nL/min. A 184 min gradient was carried out with a 10 min washing period diverted to waste after the pre-column (100% solvent A for the first 10 min followed by a gradient to 98% solvent A, v/v, at 15 min) to allow for removal of any residual salts. Following the washing period, the gradient was increased to 35% B by 125 min, followed by an increase to 90% B by 140 min and held for 9 min before returning to the initial conditions. Tandem spectra were acquired using a data-dependent scanning mode in which one full MS scan (m/z 300–2000) was acquired on the Orbitrap at a resolution of 60,000. Maximum injection time was 1 s. Data dependent MS/MS scans were acquired for the five most intense ions from the full scan using an isolation width of 2 m/z, an activation time of 30 ms, a 30% normalized collision energy, and a maximum injection time of 150 ms. 

To improve the detection of phosphopeptides, an additional data-dependent algorithm in the Xcalibur software was enabled in which the presence of a predominant phosphate neutral loss (97.97, 48.99, and 32.66 m/z units) would trigger the acquisition of an MS/MS/MS [32]. To allow collection of MS/MS spectra from lower abundance peptides, former target ions selected for MS/MS were dynamically excluded with a list length of 150 ions and a time of 60 s. “Monoisotopic precursor selection” was enabled and charge states of 1+ and >4+ were excluded from consideration for MS/MS. The instrument was operated in preview mode to allow for concurrent collection of MS/MS spectra during the Orbitrap scan. General mass spectrometric conditions were as follows: spray voltage, 2.2 kV; no sheath and auxiliary gas flow; ion transfer tube temperature, 200°C; and tube lens voltage 80 V. 

### Database searching, filtering, and false-discovery rate determination

 Methods were similar to database searching strategies previously described [28], with minor modifications. Thermo RAW files were transformed into DTA files using the ScanSifter algorithm (Vanderbilt University), then searched by TurboSEQUEST v.27 (rev. 12) algorithm (Thermo Electron) against the mouse/human subset of UniRef100 database, which was reversed to calculate the false-discovery rate. The searches were performed allowing the following differential modifications: +57 on cysteine (for carboxyimidomethylation from iodoacetamide), +16 on methionine (oxidation), and +80 on Ser, Thr, or Tyr (phosphorylation). The MS/MS/MS spectra were also searched for a -18 mass shift on Ser, Thr, or Tyr to account for the loss of water, which results from the neutral loss of phosphoric acid. Peptide and fragment ion tolerances were set to 2.5 Da and 1.0 Da, respectively. 

 False-discovery rate was then estimated from peptide matches to the reverse sequences multiplied by two and divided by the total number of peptide hits. Peptides retrieved from database search algorithms with 5% false-discovery rate were further filtered by three additional steps. First, the score threshold was set depending on peptide category. For charge 1 peptides, peptides were chosen with xcorr greater than or equal to 1, RSp less than or equal to 5, and Sp greater than or equal to 350; for charge 2 peptides, threshold was set at xcorr 1.8, RSp 5, and Sp 350; for charge 3 peptides, threshold was set at xcorr 2.5, RSp 5, and Sp 350. Secondly, only peptides with at least a tyrosine and a phosphorylation were retained. Finally, of the remaining peptide hits, only pTyr-containing peptides with at least one of three additional criteria were retained: (1) at least two matching spectra had xcorr values above high threshold (3.3 for charge of 3, 2.2 for charge of 2, and 1.5 for charge of 1), (2) the pTyr site was independently identified from two or more distinct peptides, or (3) the pTyr site had been independently identified elsewhere and included in the PhosphoSite online resource of *in vivo* phosphorylation sites, version 1.5 (http://www.phosphosite.org), maintained by Cell Signaling Technology, Inc. 

All original mass spectrometry data from the xenograph tumor experiments has been made publically available through the Chorus online mass spectrometry data resource (https://chorusproject.org/).  With a Chorus user account, any subset of these raw data can be downloaded or viewed online. Alternatively, these data can be downloaded directly as a 2.8 GB file without registering at: https://chorusproject.org/anonymous/download/experiment/-2997969850554343767).

### Antibodies and immunoblotting

 Cell samples were collected after 24 h of dasatinib exposure. Xenograft tumor samples were collected 4 h or 24 h following the final administration of dasatinib. Tumors were snap frozen in liquid nitrogen, then homogenized and diluted to 1 µg/µl in lysis buffer. Between 20 and 40 µg of total protein from each sample was loaded into 7.5-12% SDS PAGE gels and resolved by electrophoresis prior to transferring to PVDF membranes (PerkinElmer). Membranes were blocked overnight at 4°C in tris-buffered saline 0.1% Tween-20 (TBST) containing 5% w/v non fat dry milk powder. Subsequently, membranes were immunoblotted with antibodies to p-Src Y416 (Cell Signaling #6943S), total Src (Cell Signaling #2107), p-PKCδ Y313 (Cell Signaling #2055S), total PKC (Cell Signaling #2058), p-RPTP Y789 (Cell Signaling #4481S), total RPTP (Upstate #07-472), β-actin (Cell Signaling #4967), and β-tubulin (Cell Signaling #2128). Probing occurred for 1 hr at room temperature with antibodies diluted as suggested by suppliers in TBST with 3% bovine serum albumin (BSA) followed by incubation for 1 hr at room temperature with the species-appropriate horseradish peroxidase-conjugated secondary antibody (Jackson ImmunoReserach) diluted 1:5000 in TBST containing 3% BSA. Western Lightning^TM^ Plus-ECL (PerkinElmer) substrate was used for detection on a Xenogen IVIS 200. Densitometry was conducted using Living Image 3.2 software (Caliper).

### Spheroid growth analysis

 Serial microscopic images of cell spheroids from 0, 24, 48 and 72 h post dasatinib treatment were imported into the ImageJ software package (NIH). Images were automatically converted to binary and holes in the binary image were closed using the Fill Holes function in ImageJ. The Wand Selection tool was used to segment all connected pixels from the spheroid, and the number of pixels in this area was recorded. Total area for each spheroid was normalized to the 24 h time point.

### Immunohistochemistry

 Immediately following sacrifice, tumor samples of excised tumor were fixed in 10% formalin for 24 h and transferred to 70% ethanol. Samples were then processed for paraffin embedding and sectioned prior to immunostaining. Following sectioning, tissue samples were deparaffinized, rehydrated, and antigen retrieval step was conducted using a citrate buffer (pH 6.0) solution applied for 20 minutes at 105 °C followed by 10 minutes at room temperature. The samples were then treated with 3% H_2_0_2_ to eliminate endogenous peroxidase activity. The sections were subsequently blocked with a serum-free protein blocking reagent for 20 minutes. Serial sections were incubated overnight with a 1:100 dilution of p-PKCδ pY313 (Cell Signaling #2055S) antibody or p-SHIP2 Y986/987 (Cell Signaling #2008). Detection of primary antibodies utilized Envision + Systems (Dako) with a horseradish peroxidase labeled polymer (30 min incubation) followed by addition of DAB substrate (5 min incubation). Stained samples were imaged at 40x magnification and analyzed for expression of histological markers. 

## Results

### The phosphotyrosine proteome of cultured HCT-116 human colorectal cells

 We previously conducted a comprehensive analysis of the phosphotyrosine proteome of Src- transformed mouse fibroblasts *in vitro* [28]. The purpose of this study was to perform a similar analysis to characterize the pY proteome of the human CRC cell line and then extend the approach to search for potential pY biomarkers of dasatinib-responsiveness in HCT-116 xenograft tumors. 

**Figure 1:**
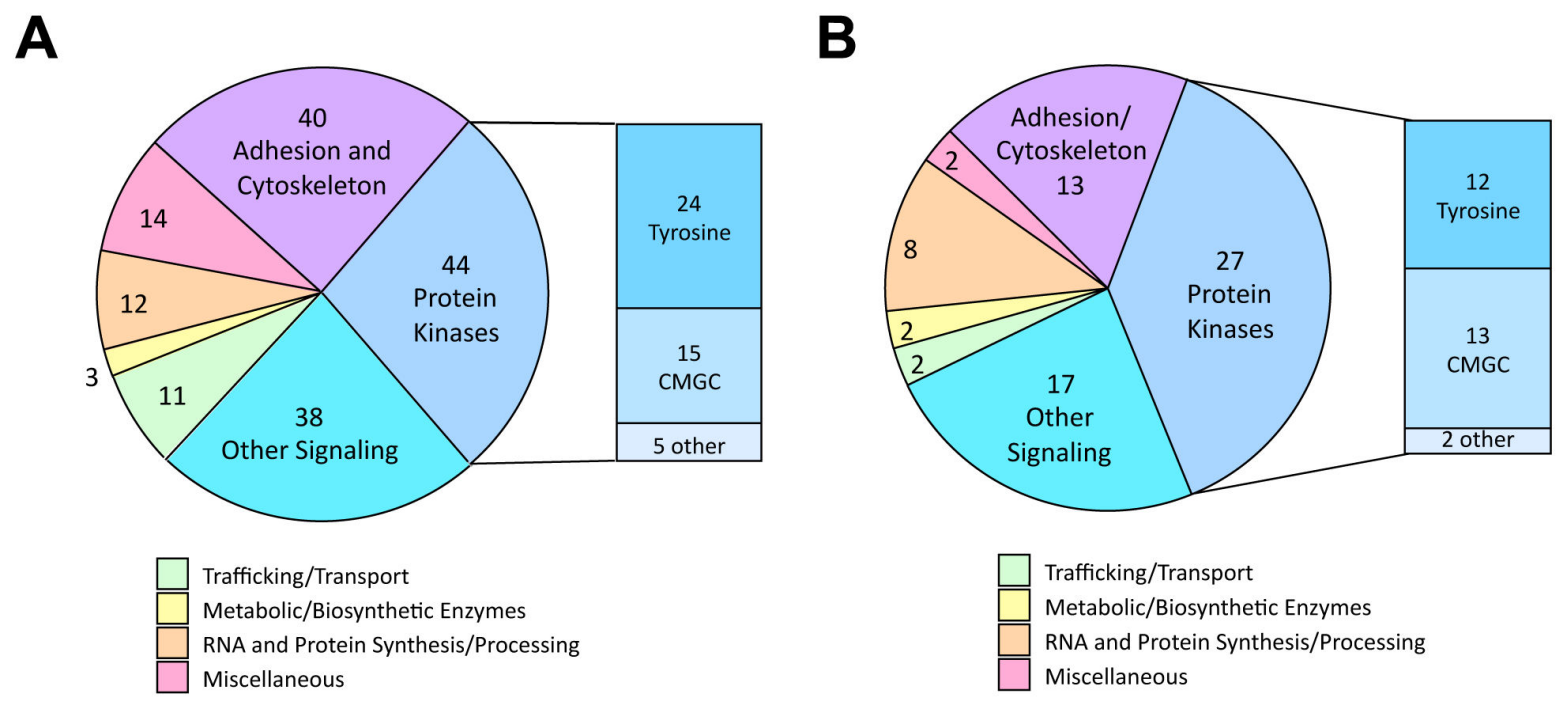
Distribution by protein functional class of pY sites readily identified in cultured HCT-116 cells and HCT-116 xenograft tumors using shotgun phosphoproteomics. In cultured HCT-116 cells, 162 pY sites were identified that represent 116 distinct proteins (**A**). Proteins classified under the broad categories of Adhesion and Cytoskeleton, Protein Kinases, and Other Signaling account for approximately three-quarters of the total. Of the pY sites on protein kinases, the vast majority are on conventional tyrosine kinases and "CMGC Group" protein kinases that includes CDKs, ERK/MAPKs, GSK-3, and other dual-specificity kinases. See Table 1 and Table 2 for a list of the most frequently identified sites by spectral count in HCT-116 cells. All 162 sites are presented in Table S1 along with key information including UniProt entry number, phosphopeptides detected in the LC-MS/MS analysis, and total spectral IDs. In HCT-116 xenograft tumors, 71 pY sites were identified that represent 58 distinct proteins (**B**). Retention criteria for the sites were 7 or more spectral IDs in tumors and detection also in HCT-116 cultured cells. The 71 pY sites represent 58 distinct proteins. Tumor sites meeting the retention criteria have a functional distribution very similar to that of sites identified in cultured cells. See Table 3 and Table 4 for a list of top sites by spectral count in HCT-116 xenografts. All 71 sites are presented in Table S2 along with key information including UniProt ID number, phosphopeptides detected in the LC-MS/MS analysis, and the number of spectral IDs from untreated *versus* dasatinib-treated tumors.

Initially, we sought to characterize the pY proteome of cultured HCT-116 cells, in the absence of dasatinib treatment. From 15 independent LC-MS/MS runs (5 biological replicates, each supported by 3 technical replicates), 249 distinct pY peptides were identified. These peptides represent 162 distinct pY sites on 116 different proteins. Criteria for peptide retention included high-threshold xcorr value and identification in > 1 biological replicate. Table S1 shows the retained peptides grouped according to a functional classification, pY site amino acid position, number of independent identifications (spectral counts), and highest xcorr values. The list of retained pY peptides and spectral count data provides a global view of pY signaling activities of HCT-116 cells under the conditions of monolayer cell culture. About half of the pY sites listed represent protein kinases (44/162) and other signaling proteins (38/162), while another 25% of the sites are on proteins associated with cell adhesion and the actin cytoskeleton (40/162) (Figure 1A). 

 Of the protein kinase pY sites, we identified 24 sites on 14 different tyrosine kinases and 15 sites on 14 different CMGC kinases. CMGC kinases (a broad classification group that includes CDKs, ERK/MAPKs, GSK-3, and families of dual-specificity kinases) were prominent among the most frequently identified HCT-116 sites (Table 1). CDK1 pY15 was the most detected site with 146 spectral counts. A kinase domain activation loop site (pY1234) on MET, hepatocyte growth factor receptor tyrosine kinase, was the second most frequently identified site in the HCT-116 cultured cell profile with 74 spectral counts. Other top-ranked sites on CMGC kinases (including PRP4, ERK2, DYRK1, and GSK-3) also reside in the kinase domain activation loops where phosphorylation indicates the activated state. We identified a number of sites in HCT-116 cells that were not observed in our previous extensive analysis of MEFs [28], Table 2.

**Table 1:** All phosphotyrosine sites most frequently identified in HCT-116 cultured cells.

**Protein**	**Functional Class**	**Site**	**IDs**
CDK1	Protein Kinase: CMGC	Y15	146
MET	Protein Kinase: Tyrosine	*Y1234	74
PRP4	Protein Kinase: CMGC	*Y849	69
CDCP1	Adhesion: Cell/ECM	Y707	47
Paxillin	Adhesion: Cell/ECM	Y118	41
ERK2	Protein Kinase: CMGC	*Y187	36
SHB	Other Signaling	Y246	32
Annexin A2	Other Signaling	Y24	29
DYRK1A	Protein Kinase: CMGC	Y321	26
SHB	Other Signaling	Y268	24
LAP2/Erbin	Other Signaling	Y1104	24
Catenin δ-1	Adhesion: Cell/Cell	Y228	24
GSK-3	Protein Kinase: CMGC	*Y279	22

* Site in protein kinase domain activation loop

Abbreviations: CDCP1, CUB domain-containing protein 1; CDK1, Cell division control protein 2 homolog; DYRK1A, Dual specificity tyrosine-phosphorylation-regulated kinase 1A; ERK2, Mitogen-activated protein kinase 1; GSK-3, Glycogen synthase kinase-3 (alpha and beta); MET, Hepatocyte growth factor receptor; PRP4, Serine/threonine-protein kinase PRP4 homolog; SHB, SH2 domain-containing adaptor protein B.

**Table 2:** Phosphotyrosine sites not found in the previous extensive analysis of cultured MEFs (28)

**Protein**	**Functional Class**	**Site**	**IDs**
CDCP1	Adhesion: Cell/ECM	Y707	47
DCBLD2	Miscellaneous	Y750	19
PKCδ	Protein Kinase: AGC	Y313	18
FAT1	Adhesion: Cell/Cell	Y4244	16
MST1R/RON	Protein Kinase: Tyrosine	*Y1238	15
Annexin A2	Other Signaling	Y30	14
DCBLD2	Miscellaneous	Y732	14
TYK2	Protein Kinase: Tyrosine	Y292	13
FYN	Protein Kinase: Tyrosine	**Y213	11
LYN	Protein Kinase: Tyrosine	**Y193	11

* Site in protein kinase domain activation loop

** Site in Src-family kinase SH2 domain

Abbreviations: CDCP1, CUB domain-containing protein 1; DCBLD2, Discoidin, CUB and LCCL domain-containing protein 2; FAT1, Protocadherin Fat 1; FYN, Tyrosine-protein kinase Fyn; LYN, Tyrosine-protein kinase Lyn; MST1R/RON; Macrophage-stimulating protein receptor; PKCδ, Protein kinase C delta type; TYK2, Non-receptor tyrosine-protein kinase TYK2.

### The phosphotyrosine proteome of HCT-116 xenograft tumors

 Having obtained the pY profile of HCT-116 cells in culture, the pY proteomics strategy was then employed to identify HCT-116 pY sites that are prominent in xenograft tumors and that have potential to serve as biomarkers for dasatinib responsiveness. Subcutaneous HCT-116 xenograft tumors were established in athymic nude mice and pY-enriched tryptic peptides were prepared from each of 3 tumors from both vehicle-treated mice and from mice treated 4 hours earlier with 60 mg/kg dasatinib. Each peptide preparation was analyzed through three replicate LC-MS/MS runs to obtain the tumor pY profiles. From this analysis, 71 pY sites, representing 58 different proteins, were recognized as being prominent in the xenograft tumors as well as being detected in the cultured cells (Table S2). Similar to the cultured cell profile, most of these sites were on signaling proteins (including many protein kinases) and adhesion/cytoskeleton-associated proteins (Figure 1B). Tyrosine kinases and CMGC protein kinases were again prominent in the tumor profile. Sites on CMGC kinases dominated the list of most frequently identified tumor sites including CDK1 Tyr15, and the activation loop sites on p38 MAPK, PRP4, ERK1, ERK2, and GSK-3 (Table 3). 

**Table 3:** Phosphotyrosine sites most frequently identified in untreated HCT-116 xenograft tumors: comparison to dasatinib-treated animals.

**Protein**	**Functional Class**	**Site**	**IDs (untreated, 4hr Das)**
CDCP1	Adhesion: Cell/ECM	Y707	90, 14
CDK1	Protein Kinase: CMGC	Y15	85, 80
PKCδ	Protein Kinase: AGC	Y313	72, 10
p38α MAPK	Protein Kinase: CMGC	*Y182	59, 35
PRP4	Protein Kinase: CMGC	*Y849	49, 45
ERK2	Protein Kinase: CMGC	*Y187	35, 31
ERK1	Protein Kinase: CMGC	*Y204	30, 27
RPTPα	Other Signaling	Y798	27, 1
β-actin	Cytoskeleton	Y55	22, 24
GSK-3	Protein Kinase: CMGC	*Y279	19, 22
Annexin A2	Other Signaling	Y30	19, 5
Histone H4	Miscellaneous	Y89	19, 17

* site in activation loop of protein kinase domain

Abbreviations: CDCP1; CUB domain-containing protein 1; CDK1, Cell division control protein 2 homolog; ERK1, Mitogen-activated protein kinase 3; ERK2, Mitogen-activated protein kinase 1; GSK-3, Glycogen synthase kinase-3 (alpha and beta); p38α MAPK, Mitogen-activated protein kinase 14; PKCδ, Protein kinase C delta type; PRP4, Serine/threonine-protein kinase PRP4 homolog; RPTPα, Receptor-type tyrosine-protein phosphatase alpha.

 CDCP1 pY707 (90 spectral counts), PKCδ pY313 (72 spectral counts), and PKCδ pY334 (14 spectral counts) were among the most frequently identified sites in untreated xenograft tumors. Dasatinib treatment greatly reduced the identification frequency of all three sites (Table 3, Table 4), consistent with their being targets of Src-family kinases. In contrast, the sites on CMGC kinases (not considered to be phosphorylated by dasatinib-sensitive kinases) were identified at similar frequencies in untreated and dasatinib-treated tumors. Other apparent dasatinib-responsive sites among the most frequently identified in vehicle-treated tumors included pY798 on RPTPα and pY30 on Annexin A2 (Table 3). RPTPαpY798 was identified in 27 spectra from untreated tumors, but only once in dasatinib-treated samples. 

**Table 4:** Other notable dasatinib-responsive phosphotyrosine sites identified in untreated HCT-116 xenograft tumors: comparison to dasatinib-treated animals.

**Protein**	**Functional Class**	**Site**	**IDs (untreated, 4hr Das)**
RAIG-1	Other Signaling	**Y347**	**15, 1**
PKCδ	Protein Kinase: AGC	**Y334**	**14, 0**
SHC1	Other Signaling	**Y427**	**13, 0**
α-enolase	Metabolic Enzyme	**Y44**	**13, 3**
SHIP2	Other Signaling	**Y986**	**11, 0**

Abbreviations: PKCδ, Protein kinase C delta type; PRP4, Serine/threonine-protein kinase PRP4 homolog; RAIG-1, Retinoic acid-induced protein 3; SHC1, SHC-transforming protein 1; SHIP2, Phosphatidylinositol-3,4,5-trisphosphate 5-phosphatase 2.

### Immunoblotting validates dasatinib responsiveness of PKCδ pY313 and RPTPα pY798 sites in HCT-116 xenograft tumors

**Figure 2:**
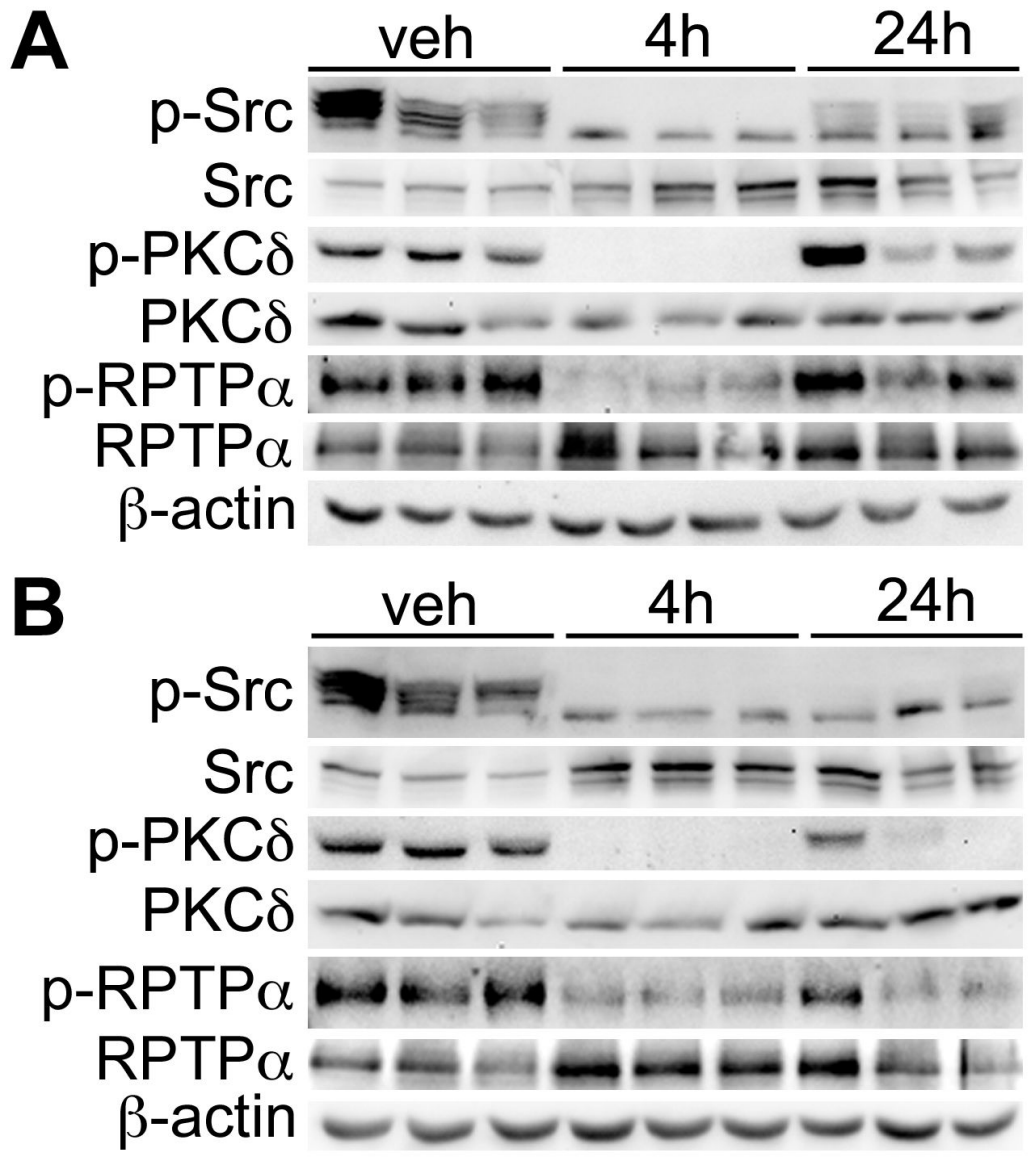
Immunoblot analysis of HCT-116 xenograft tumors to validate pY site identifications as potential biomarkers of dasatinib response. Immonoblot analysis of PKCδ pY313 and RPTPα pY798 in xenograft tumors in relation to Src pY416 after a single pharmacological dose (**A**) or 5 daily doses of dasatinib (**B**). Xenograft tissues were evaluated 4 h and 24 h following dasatinib exposure. Both PKCδ pY313 and RPTPα pY798 correlate with Src inhibition in HCT-116 xenografts. In addition to a single dose, steady-state treatment regimens (**B**) resulted in prolonged Src inhibition as measured 24 h after dasatinib exposure and correlated with decreased levels of PKCδ pY313 and RPTPα pY798.

 Of the most prominent dasatinib-responsive pY sites identified from the phosphoproteomic analysis of HCT-116 xenograft tumors (Table 3, Table 4), phospho-specific antibodies are commercially available for PKCδ pY313, SHIP2 pY986/987 and RPTPα pY798. These antibodies were used to validate dasatinib responsiveness of these sites relative to Src inhibition (determined by using a phosphospecific antibody against the Src pY416 autophosphorylation site). For both the PKCδ pY313 and RPTPα pY798 sites, immunoblotting with phospho-specific antibodies indicated a marked decrease in phosphorylation in 4 h dasatinib-treated tumors concomitantly with Src inhibition (Figure 2A). The phosphorylation status of these residues returned to near baseline levels 24 hours post-treatment, suggesting the durability of Src inhibition in this model was transient with a single administration. 

To evaluate the effect of dasatinib treatment duration on the durability of Src inhibition, HCT-116 tumors treated with 5 daily doses of dasatinib were assessed by western blot analysis to determine the response of identified residues under steady-state drug administration. Similar to tumors treated with a single pharmacological dose, these tumors exhibited coordinate reductions in Src pY416, RPTPα pY798, and PKCδ pY313 observed 4 h following the final administration of dasatinib (Figure 2B). However, unlike the response seen after a single dasatinib dose, recovery of p-Src, p-RPTPα, and PKCδ to baseline levels was incomplete 24 h following the final drug administration suggesting a more durable inhibition following repeated dasatinib administration. 

### IHC analysis

**Figure 3:**
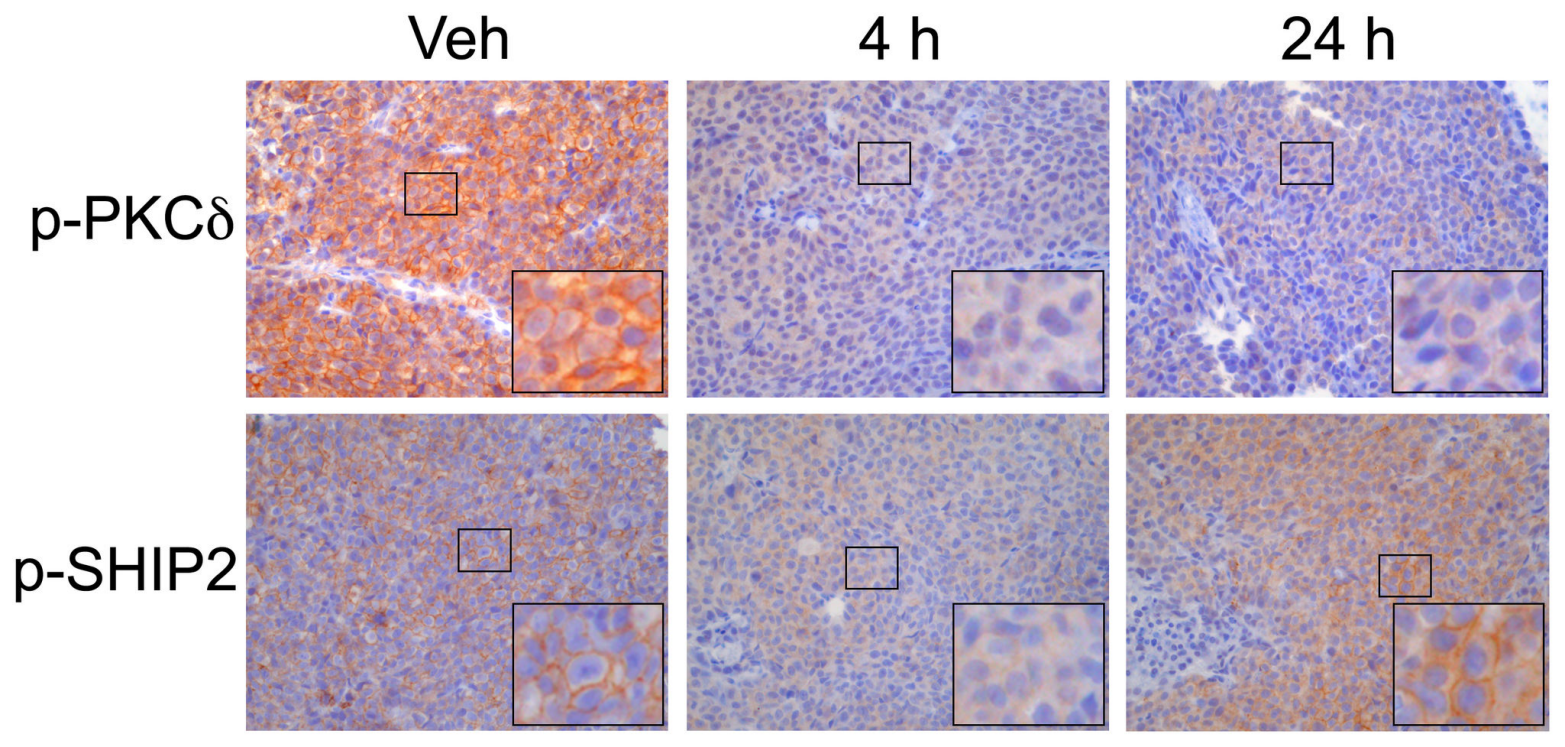
IHC analysis of HCT-116 xenograft tumors treated with a single dose of dasatinib. Representative 40x magnification images show robust cytosolic and membranous staining for PKCδ pY313 (top panels). At 4 h after dasatinib treatment, both cytosolic and membranous PKCδ pY313 staining are attenuated. By 24 h following dasatinib administration, PKCδ pY313 begins to rebound in both the cytosolic and membranous compartments. Similarly, p-SHIP2 staining (bottom panels) shows primarily membranous localization with minor cytosolic staining in vehicle treated controls. After 4 h dasatinib treatment decreased cytosolic and membranous staining is observed. Like PKCδ pY313, SHIP2 pY986 levels return to baseline levels 24 h following treatment.

In addition to immunoblotting, PKCδ pY313 was evaluated in formalin fixed, paraffin-embedded tumor tissues. HCT-116 xenografts treated with dasatinib (60 mg/kg) or vehicle were evaluated 4 h and 24 h after a single pharmacological dose. IHC analysis of PKCδ pY313 showed robust membranous and cytosolic staining in vehicle treated mice (Figure 3). Analysis of tissues collected 4 h after dasatinib exposure demonstrated greatly attenuated cytosolic p-PKCδ staining. Similar to observations with immunoblotting, PKCδ pY313 levels rebounded 24 hours following dasatinib administration. Similar results were obtained using an antibody recognizing the SHIP2 pY986 site (Figure 3), also identified as a dasatinib-responsive site in our study. 

### In vitro immunoblotting

**Figure 4:**
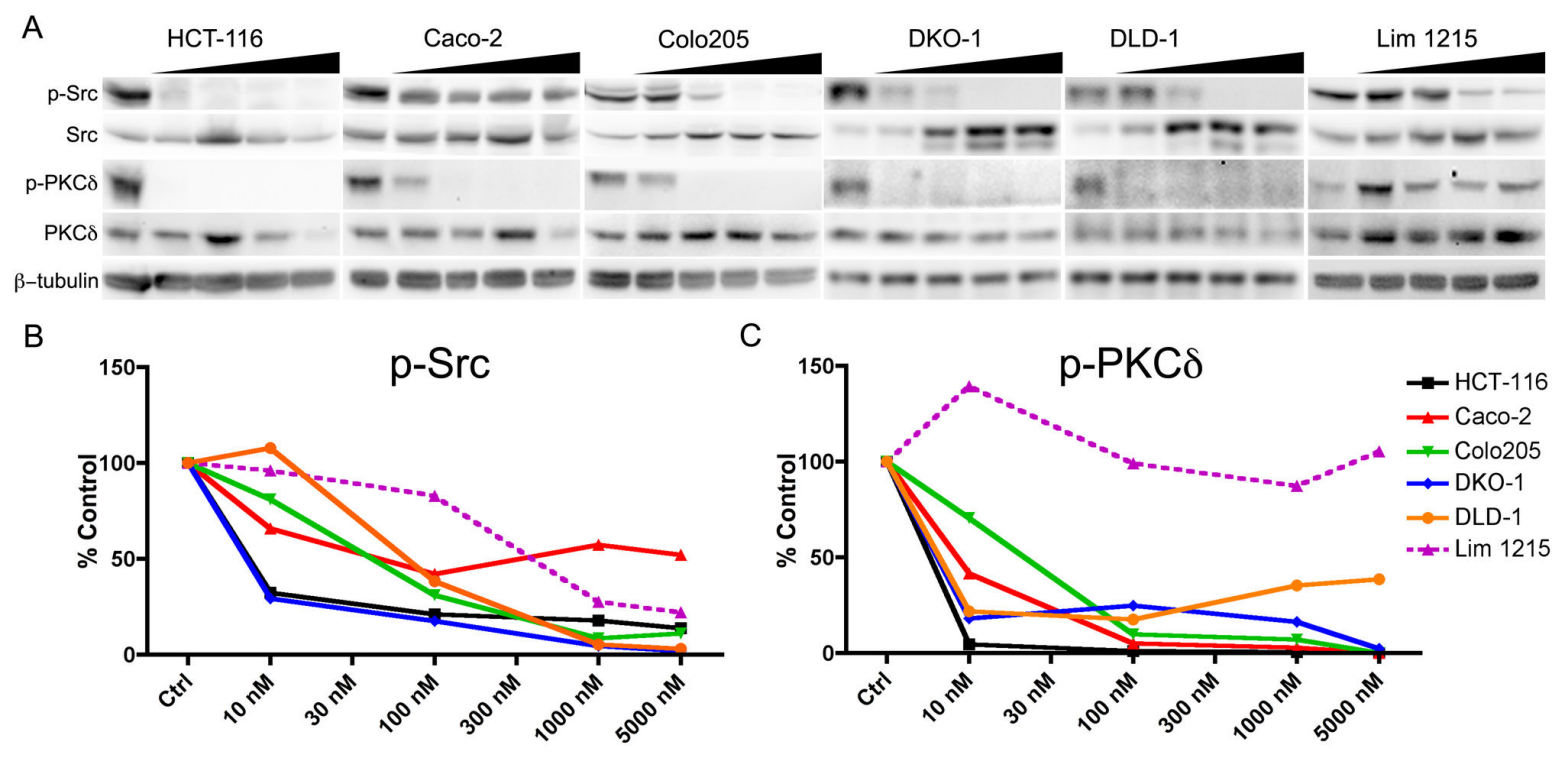
Immunoblot analysis of a panel of CRC cell lines further validate PKCδ PY313 as a potential biomarker of dasatinib response. The relationship between Src inhibition and PKCδ pY313 was evaluated as a function of dasatinib exposure (0, 10, 100, 1000, 5000 nM) in HCT-116, Caco-2, Colo204, DKO-1, DLD-1, and Lim1215 cell lines (A). Densitometry showed a concentration-dependent decrease in Src pY416 levels indicative of Src inhibition (**B**) in all cell lines except Lim1215. In cell lines were Src pY416 was reduced, a similar concentration dependent decrease in PKCδ pY313 was observed (**C**).

 To evaluate the relationship between Src inhibition and PKCδ pY313 more broadly, levels of Src pY416 and PKCδ pY313 were evaluated as a function of dasatinib exposure in a panel of CRC human cell lines *in vitro* including HCT-116, Caco-2, Colo205, DKO-1, DLD-1, and Lim1215. In all cell lines evaluated except Lim1215, dasatinib exposure reduced Src pY416 in a concentration-dependent manner (Figure 4) indicative of Src inhibition. In all cell lines where Src was effectively inhibited, a similiar conentration-dependent reduction in PKCδ pY313 was also observed. In Lim1215 cells, Src pY416 appeared more recalcitrant to dasatinib exposure and PKCδ pY313 did not correlate with Src inhibition.

### Reduction of PKCδ pY313 by dasatinib correlates with reduced spheroid growth

**Figure 5:**
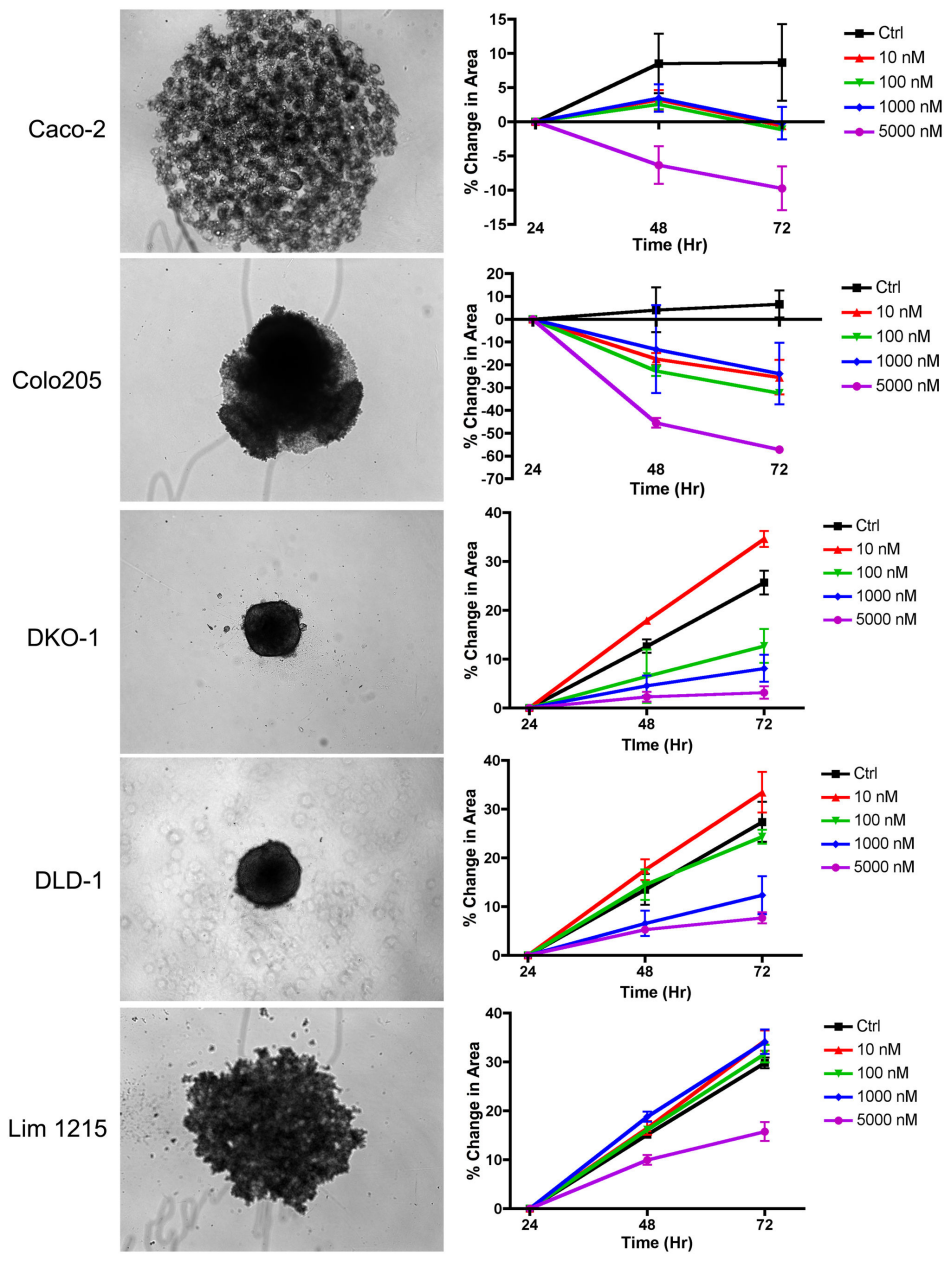
Cellular proliferation analysis by changes in spheroid size in response to dasatinib treatment. Representative microscopy images (left panels) of Caco-2, Colo205, DKO-1, DLD-1, and Lim1215 spheroid cultures are shown. When grown in the presence of dasatinib, all cell lines which resulted in potent reduction Src pY416 and PKCδ pY313 (see Figure 4) also resulted in concentration-dependent inhibition of spheroid growth (right panels). Lim1215 cells which did not exhibit reduced levels of Src pY416 and PKCδ pY313 in response to dasatinib showed little change in growth due to dasatinib exposure.

 To evaluate the relationship between PKCδ pY313 and proliferation, Caco-2, Colo205, DKO-1, DLD-1 and Lim1215 cells were propagated as spheroid cultures in the presence of various concentrations of dasatinib (Figure 5). Interestingly, in all cell line cultures for which dasatinib resulted in potent Src inhibition and reduced levels of PKCδ pY313 (Caco-2, Colo205, DKO-1, DLD-1, Figure 4) dasatinib exposure resulted in a concentration dependent effect on spheroid growth. However, Lim1215 cells which were recalcitrant to the dasatinib-mediated reduction of Src pY416 and PKCδ pY313, showed little sensitivity to dasatinib.

## Discussion

 In CRC, Src family kinases display elevated activity that increases with disease progression and has been linked to poor clinical prognosis. Dasatinib, the most clinically studied Src inhibitor, is an effective cytostatic agent utilized as a therapy for various cancers, including CRC. Reliable biomarkers of dasatinib response hold great potential to guide both preclinical therapeutic development and patient care. 

 HCT-116 colonic carcinoma cells grow rapidly in monolayer culture as adherent, closely packed polygonal cells and readily give rise to epithelioid tumors when injected subcutaneously into nude mice [33]. Tumorigenicity of HCT-116 cells has been linked to an activating mutation in the *KRAS* gene [34]. To assess the pY proteome of HCT-116 cells, we carried out LC-MS/MS analysis of tryptic peptides enriched for pY by immunoaffinity purification [27]. Extensive application of this shotgun approach can provide a global view of pY signaling pathways within cells, with spectral counts reflective of pY site relative abundance [28]. Utilizing this phosphoproteomics approach, we identified 249 distinct pY sites in the *in vitro* HCT-116 proteome. The functional distribution of the represented proteins is quite similar to the profile obtained from previous analysis of nontransformed mouse embryo fibroblasts (MEFs), further validating this phosphoproteomic approach [28]. Many identified signaling proteins are members of the CMGC kinase family. The pY sites on these kinases arise from autophosphorylation and/or phosphorylation by dual-specificity protein kinases, rather than by conventional tyrosine kinases such as Src. Many of these sites also reside in the kinase domain activation loops, where phosphorylation indicates the active state. The presence of one such site on MET pY1234 in the profile may reflect its activation by cell adhesion [35] rather than by growth factor. The findings of CDK1 pY15 as the most frequently identified HCT-116 cell site, and the strong representation of kinase domain activation loop sites on other CMGC kinases and MET receptor tyrosine kinase, mirror results from our analysis of mouse fibroblasts [28]. Given their activation by kinases other than Src family members, these are unlikely to be relevant as specific markers of dasatinib activity.

 Notable among the other most frequently identified HCT-116 cell pY sites is pY707 on CUB-domain-containing protein 1 (CDCP1). This site has the distinction of being the most frequently identified site not previously found in our MEF analysis (Table 2). CDCP1, also known as Trask (Transmembrane and associated with Src kinases), is a membrane-spanning glycoprotein encoded by a gene commonly over-expressed in CRC [36,37]. CDCP1 is phosphorylated by Src-family kinases upon loss of cell/ECM adhesion [38,39], which promotes formation of a signaling complex consisting of CDCP1, Src, and PKCδ [40]. The Src/CDCP1/PKCδ signaling complex is implicated in anchorage-independent cell survival and cell migration/invasion [41,42]. In complex with CDCP1, Src also phosphorylates PKCδ [40]. Thus it is notable that PKCδ pY313 is also among the most frequently identified HCT-116 cell pY sites not previously found in the MEF analysis (Table 2). These findings indicate that the Src/CDCP1/PKCδ signaling complex is strongly activated in HCT-116 cells.

 Further indication of the capacity of pY proteomics to reveal distinct signaling activities in HCT-116 cells are pY sites readily detected on tyrosine kinases EGFR (pY1197), macrophage-stimulating protein receptor (MST1/RON (pY1238)), and the JAK-family member TYK2 (pY292). These tyrosine kinase sites were prominent in the HCT-116 cell profile with 9, 15, and 13 spectral IDs, respectively, but none were detected in the previous MEF analysis [28]. An intriguing observation is that equivalent pY sites in the SH2 domains of two different Src-family kinases (FYN pY213 and LYN pY193) are among the most frequently identified HCT-116 cell sites not found MEFs (Table 2). It is likely that phosphorylation of these sites results in kinase activation due to impairment of SH2 domain function, as was shown for the equivalent site on Src [43]. 

 Phosphoproteomics of HCT-116 xenograft tumors returned results simliar to those seen in cultured HCT-116 cells. Differential analysis of these samples revealed 71 distinct pY sites conserved from cultured cells to xenograft tumors. Similar to *in vitro* findings, many sites were identified on active signaling proteins, including many protein kinases. Notably, CDCP1 pY707 and two distinct sites on PKCδ (pY313 and pY334) were again identified. The ready identification of these three sites suggests high activity of the Src/CDCP1/PKCδ signaling complex in the HCT-116 tumors, indicating that observed pY hits on PKCδ may indeed serve as surrogates of Src activity and inhibition.

Additional dasatinib-responsive sites found in xenograft tumors included RPTPα Y798 and Annexin A2 pY30. RPTPα Y798 resides in the cytoplasmic tail near the C-terminus, is a Src target [44], and has been linked to cytoskeletal reorganization in response to integrin engagement [45]. Annexin A2 pY30 also known as calpactin I, was one of the earliest recognized Src substrates [46] although the functional significance of Y30 phosphorylation is still uncertain. 

Western blot confirmation of PKCδ pY313 and RPTPα Y798 as potential biomarkers illustrated that the effect of dasatinib on these sites was highly transient. The lack of durability in Src inhibition is consistent with the 3-5 h half-life reported for dasatinib *in vivo* [47]. Assessment of these proteins under an extended treatment protocol (treatment daily for 5 days) also revealed evidence indicative of a return toward baseline levels 24 h following the final drug administration, though significantly improved inhibition was observed. 

Our results also suggest that the Src autophosphorylation site pY416 and the pY313 site on PKCδ are correlated in CRC cells that are responsive to dasatinib. This was observed in 4 human CRC cell line models. Importantly, though Src pY416 was inhibited at elevated dasatinib concentrations in Lim1215 cells, PKCδ pY313 did not correlate with p-Src in this model and the lack of an effect on PKCδ pY313 predicted a lack of response. These results suggest that measuring Src pY416 levels alone may be insufficient to predict patient responses to dasatinib and that incorporating PKCδ pY313 may be a complementary measure of treatment response.

 In summary, we have utilized a shotgun pY proteomics approach to identify candidate pY biomarkers of dasatinib response in HCT-116 cells and xenograft tumors. Several dasatinib-responsive pY sites representing important signaling proteins were identified in both *in vitro* and xenograft tumor screens and were subsequently validated in xenograft tumors using phosphospecific antibodies. These studies suggest that a subset of these potential markers, RPTPα pY798 and PKCδ pY313 in particular, represent promising biomarkers of response to dasatinib and may represent sensitive measurable downstream surrogates of Src inhibition and overall response in CRC.

## Supporting Information

Table S1 **Phosphotyrosine peptides from HCT-116 cultured cells.**
(XLS)

Table S2 **Phosphotyrosine peptides from HCT-116 tumors: untreated vs. dasastinibReferences.**
(XLS)
